# Seasonal Influenza Vaccine Acceptance among Pregnant Women in Zhejiang Province, China: Evidence Based on Health Belief Model

**DOI:** 10.3390/ijerph14121551

**Published:** 2017-12-11

**Authors:** Yu Hu, Ying Wang, Hui Liang, Yaping Chen

**Affiliations:** Institute of Immunization and Prevention, Zhejiang Center for Disease Control and Prevention, Hangzhou 310000, China; ywang@cdc.zj.cn (Y.W.); hliang@cdc.zj.cn (H.L.); ypchen@cdc.zj.cn (Y.C.)

**Keywords:** seasonal influenza vaccine, acceptance, pregnant women, health belief model

## Abstract

*Background:* Reasons for acceptance of seasonal influenza vaccine (SIV) vaccination among pregnant women in China are poorly understood. We assessed the intention to accept SIV among pregnant women in Zhejiang province, by using a self-administrated structured questionnaire developed on the basis of health belief model (HBM). *Methods:* From 1 January to 31 March 2014, pregnant women with ≥12 gestational weeks who attended antenatal clinics (ANCs) at public hospitals in 6 out of 90 districts were surveyed using a self-administered questionnaire that covered knowledge, attitudes, and beliefs related to SIV vaccination and influenza infection. We examined the associations between the acceptance of SIV vaccination and the demographic factors and HBM constructs using the logistic regression model, calculating the adjusted odds ratio (AOR). *Results:* Of the 1252 participants, 76.28% were willing to receive the SIV vaccination during their current pregnancy. High levels of perceived susceptibility of influenza (AOR = 1.75 (95%CI: 1.36–2.08)), high levels of perceived severity of influenza (AOR = 1.62 (95%CI: 1.25–1.95)), high level of perceived benefits of vaccination (AOR = 1.97 (95%CI: 1.76–2.21)), and high levels of cues to action were positively associated with the acceptance of SIV vaccination among pregnant women (AOR = 2.03 (95%CI: 1.70–2.69)), while high level of perceived barriers of vaccination was a negative determinant (AOR = 0.76 (95%CI: 0.62–0.94)). *Conclusions:* Poor knowledge and negative attitude towards SIV were associated with the poor acceptance of SIV. Health providers’ recommendations were important to pregnant women’s acceptance of SIV. Health education and direct communication strategies on SIV vaccination and influenza infection are necessary to improve the acceptance of SIV vaccination among pregnant women.

## 1. Introduction

Influenza is an important cause of morbidity and mortality worldwide, and pregnant women are at increased risk of severe complications compared with the non-pregnant population [1]. For example, the mortality among pregnant women from influenza and pneumonia during the 2009 influenza A (H1N1) pandemic was 2- to 3-fold higher than that in the non-pregnant population, and women who died were more likely to be pregnant [2]. Children under 6 months old are also vulnerable, bearing the highest rate of influenza-related hospitalization [3].

Seasonal influenza vaccine (SIV) vaccination is the most effective strategy for preventing the influenza infection and reducing the influenza-related complications. SIV vaccination during pregnancy provides benefits to both a pregnant woman and her newborn [4]. Previous studies have found that maternal SIV vaccination is effective at preventing laboratory-confirmed influenza in infants up to 6 months of age, who are still ineligible for SIV vaccination as no SIV has been licensed for use in children under 6 months old [5,6]. Furthermore, current evidence suggests that maternal SIV vaccination can offer the secondary protection to infants for at least the first 6 months of their lives through the transmission of the placental transfer antibody [7]. 

Pregnant women in any trimester have been considered as the highest priority group for SIV vaccination by the World Health Organization (WHO) [8]. Also, the Advisory Committee on Immunization Practice of the United States recommended annual vaccination of SIV for pregnant women [9]. In 2014, the Chinese Advisory Committee on Immunization Practice (CACIP) updated the guidelines for the application of SIV, which recommended the SIV vaccination for pregnant women as a high priority group. 

Zhejiang province is located in east China and is one the most populous and developed province in China, with a total area of 104,141 km^2^ and a population of 70 million residents. According to data of the national notifiable disease surveillance system, the incidence of influenza-related illness was 26.09 per 100,000 in 2016 in Zhejiang province. At present, SIV has not been included in the Chinese expanded program on immunization (CEPI) schedule for pregnant women and children. The SIV is a category II (parent-pay) vaccine and the vaccination is voluntary in China. 

Data on the coverage of SIV among pregnant women in Zhejiang province are limited; however, the coverage rates of SIV among adults and children aged 6 months to 3 years old were less than 1% in recent years [10]. There is a pending need to understand the reasons for low uptake of SIV, and the knowledge, attitude and health beliefs of pregnant women on SIV vaccination have not been investigated extensively. In this study, we aimed to identify the impact factors that were associated with the acceptance of SIV vaccination among pregnant women. Our finding may provide critical guidance for improving communications directed at pregnant women’s awareness of the benefits of SIV vaccination and concerns regarding vaccine safety. 

## 2. Methods

### 2.1. Study Setting and Subjects

This study was conducted in 6 districts (total of 90 districts in Zhejiang Province). According to the data from Zhejiang provincial bureau of statistics, the total population of Yinzhou, Dinghai, Dongyang, Changxing, Liandu and Kecheng in 2014 was 840,108, 383,859, 830,664, 628,175, 460,358 and 436,856, respectively. In each district, four public obstetric hospitals with annual number of deliveries ≥500 in 2013 were selected and in total 24 hospitals were chosen as the investigation sites. When more than four hospitals met our selection criteria in a single district, the top four with the highest annual number of deliveries were selected. Pregnant women with ≥12 gestational weeks who attended antenatal clinics (ANCs) in participating obstetric hospitals were recruited from 1 January 2014 to 31 March 2014. In this study, migrant was defined as the person who lived in a district other than their hometown (even if from the same province) but had no local registration of the current living place. 

### 2.2. Sample Size

The formula used to estimate the sample size was as follows: $N=\frac{1.96^{2}\times p\times \left(1-p\right)}{d^{2}}$. The proportion of acceptance of SIV among pregnant women was assumed to be 80% according to an approximate estimate from a study conducted in Pakistan [11]. Furthermore, a *p*-value of 0.05, the desired precision of 5% and a design effect of 2 were also used for the sample size calculation. We used these parameters to estimate the sample size to ensure a larger minimum sample size. Thus, a minimum sample size of 491 subjects would be sufficient to estimate the proportion of acceptance of SIV. Considering the feasibility of this study, the final sample size was 600 eligible pregnant women or 25 in each group for every selected hospital.

### 2.3. Enrollment Process

The enrollment period was from 1 January to 31 March 2014. The ANC clinics were usually open two to four days per week, and enrollment were only administered on days during which ANC clinics were open. Hence, the recruitment was conducted on multiple days in order to reach the sample size for each hospital. Medical staff at each selected hospital approached pregnant women who visited ANC clinics to determine eligibility. All eligible pregnant women would receive a cover letter describing the adequate details of study objectives, methods, potential risks. Once a written informed consent was obtained, participant was required to complete a survey on sites, using a self-administrated questionnaire. For each hospital, the enrollment would be ended if 50 eligible pregnant women were recruited.

### 2.4. Questionnaire

A self-administrated structured questionnaire was developed by the study team and was pilot-tested among a convenience sample of 20 pregnant women, who were interviewed to obtain the general acceptability of the questionnaire in terms of length, clarity, and question formats. The questionnaire requested demographic information including age, education, immigration status, employment status, income, number of children. Information related to the knowledge of influenza virus infection and SIV vaccination was collected. Attitudes towards SIV vaccination were based on health belief model (HBM) [12], which included five constructs that influence health behaviors namely perceptions of susceptibility, severity, barriers, benefits and cues to action. The HBM assumed that people are likely to take disease prevention behaviors (like vaccination) if they perceive that they are susceptible to the disease or the disease is severe or the behavior is beneficial or barriers are minimal. Furthermore, cues to action, such as recommendations from immunization provider or health education messages can also influence the behaviors [13]. We adapted and modified questions from the previous published literature and translated into the Chinese language [14]. Good internal consistency reliability was found for the questionnaire with the Cronbach’s α coefficient of 0.77.

The questionnaire included two statements focused on perceived susceptibility to influenza infection for both mother and infant, two on perceived severity of influenza infection for mother and infant, three on perceived barriers of SIV vaccination, three on perceived benefits of SIV vaccination, and two on cues to action. The response answers of each HBM individual item included “agree or very concerned”, “not sure or moderate concerned” and “disagree or not concerned”.

### 2.5. Outcome

First, enrolled pregnant women who reported having received a SIV vaccination or reported that they wanted to get SIV during their current pregnancy were considered as willing to accept. Second, we grouped response answers for HBM individual items into two groups: (1) agree, or (2) not sure or disagree. Participants’ levels of concern about personal susceptibility to influenza during this pregnancy and their fetus’s susceptibility were categorized as (1) very/moderate concerned, or (2) not concerned. Third, every individual HBM item was re-coded to three levels such that higher values corresponded to a greater degree of agreement or importance as: 1 = “disagree or not concerned”; 2 = “not sure or moderate concerned”, and 3 = “agree or very concerned”. The scores of individual HBM items were combined based on conceptual similarity into HBM constructs and then summed to create new scores for each component of the HBM framework. Enrolled pregnant women were divided into tertiles by the new summed score to create three (low/moderate/high) categories for each HBM construct to facilitate the data interpretation.

### 2.6. Data Analysis

The associations between the demographic characteristics and the acceptance of SIV and the associations between the HBM items (categorized into two groups) and the acceptance of SIV were assessed by the Chi-square test. 

We employed logistic regression models to identify the HBM constructs associated with the willingness to accept SIV vaccination. Crude models included only the HBM constructs, while adjusted model included all the demographic variables. The potential independent variables with a *p* value < 0.1 in the bivariate analysis (the Chi-square test) were then included in the multivariable regression using backward likelihood ratio method. All HBM constructs were included in the logistic regression model simultaneously. Crude and adjusted odds ratios with 95%CIs for each variable were also calculated. All statistical analyses were performed with STATA MP 14.0 (Stata Corp. 2015, Stata statistical software, College Station, TX, USA).

### 2.7. Ethical Considerations

This study was approved by the Ethical Review Board of Zhejiang Provincial Center for Disease Control and Prevention (T-019-S).

## 3. Results

### 3.1. Demographic Characteristics and Acceptance of SIV Vaccination

Of the 1305 pregnant women approached, 1252 (95.94%) agreed to participate in this study (Table 1). Of the enrolled pregnant women, 766 (61.18%) were 20–30 years old, 673 (52.75%) were migrant, 755 (60.30%) had a college education background or above, 113 (9.03%) were housewives, 383 (30.59%) pregnant women’s household had a monthly income per capita over 1500 CNY, 635 (50.72%) pregnant women had no child before the current pregnancy. The proportion of acceptance of SIV vaccination among the participants was 76.28%. The demographic characteristics were significantly different between pregnant women accepting and not accepting SIV vaccination.

### 3.2. Health Beliefs and Acceptance of SIV Vaccination

Perceived susceptibility to influenza, severity of influenza, benefits and cues to action of SIV vaccination were higher among pregnant women accepting SIV vaccination than those not, while the barriers of SIV vaccination were lower among pregnant women accepting SIV vaccination than those not (Table 2). For example, of the pregnant women accepting the SIV vaccination, 65.13% perceived themselves as susceptible to influenza compared with 27.95% of the not-accepting SIV vaccination group. A greater proportion of pregnant women who accepted SIV vaccination significantly believed SIV could protect their unborn child (76.02% vs. 52.86%). A greater proportion of pregnant women who did not accept SIV vaccination held a misunderstanding that SIV vaccination was unsafe during pregnancy (29.97% vs. 14.87%). More pregnant women who accepted SIV vaccination thought receiving SIV during pregnancy would benefit her fetus and new born baby (77.28% vs. 61.95%). Pregnant women intended to accept SIV vaccination were much more likely to respond to cues to action to be vaccinated from physicians or nurse (93.09% vs. 74.41%).

### 3.3. HBM Constructs and Acceptance of SIV Vaccination

In multivariable models, high levels of perceived susceptibility of influenza (AOR = 1.75 (95%CI: 1.36–2.08)), high levels of perceived severity of influenza (AOR = 1.62 (95%CI: 1.25–1.95)), high level of perceived benefits of vaccination (AOR = 1.97 (95%CI: 1.76–2.21)), and high levels of cues to action were positively associated with the acceptance of SIV vaccination among pregnant women (AOR = 2.03 (95%CI: 1.70–2.69)), while high level of perceived barriers of vaccination was a negatively determinant (AOR = 0.76 (95%CI: 0.62–0.94)) (Table 3).

## 4. Discussion

In this study, the pregnant women receiving antenatal care at public hospitals in Zhejiang province were investigated as part of a provincial SIV vaccination program promotion. Although the WHO and the Advisory Committee on Immunization (ACIP) of the United States recommended that all pregnant women should be immunized SIV as they are the most important risk group for seasonal influenza compared to all risk groups and both pregnant women and infants will most likely benefit from the vaccination, the acceptance of SIV among pregnant women was 76.28% in 6 districts in Zhejiang province. This result was consistent with other developed countries, such as the United States [15]. 

Health belief model (HBM) theory provides a valuable framework for evaluating the knowledge and attitude towards the seasonal influenza vaccine (SIV) vaccination behavior. In this study, we found that pregnant women with low knowledge of SIV vaccination or influenza infection, such as the susceptiblity and severity of the influenza infection to them or their unborn baby, were significantly less likely to take the SIV as opposed to those with good knowledge in both descriptive analysis and multivariable analysis. Our findings were consistent with several previous studies of pregnant women conducted in Western countries. For example, Ahluwalia [16] indicated that unvaccinated respondents cited a variety of reasons for not receiving the SIV including worries that the vaccine might harm their babies (27%) or themselves (26%), by using the data from the Georgia pregnancy risk assessment and monitoring system. Similarly, the results from a cross-sectional survey of pregnant women in Pennsylvania indicated that 61% of the women reported concern on vaccine safety during pregnancy and 8% reported the belief that the SIV could cause the influenza infection [17]. Second, our findings indicated that the proportions of lack of awareness on the benefits or overestimation of the barriers of vaccination were significantly higher in the participants who did not accept the SIV during pregnancy, in both descriptive analysis and multivariable analysis. Similarly, previous studies [18,19] have also confirmed that the poor attitudes towards the benefits of the SIV were obstacles to vaccine receipt. As such, we suggested that health education programs focused on SIV and influenza infection should be emphasized and successful experience had been found in other countries or settings. One study from Australia [20] showed that the coverage of SIV increased from 30% in 2010 to 40% in 2011 after the implementation of a health educational program for maternity staff and pregnant women. Similar results were observed in Canada [21], where showed an increase in coverage of SIV from 19% in 2006 to 56% in 2007, after distributing educational pamphlets on influenza in antenatal clinics. During medical counseling of attended antenatal clinic (ANC), physicians must emphasize one of the greatest vaccine benefits, which is that it can reduce the risk of respiratory illnesses and hospital admissions for them as well as for their newborn infants up to 6 months old. Audio or visual presentations focusing safety, efficacy, and potential benefits of the SIV vaccination should be presented to pregnant women when they are waiting at the antenatal clinic for their appointment.

Our results highlighted the healthcare provider recommendations were an important cue to action for SIV acceptance among pregnant women. Previous reports had demonstrated that healthcare providers’ attitudes and beliefs around influenza vaccination clearly influence vaccine uptake. One study showed vaccination awareness campaigns aimed at obstetricians, primary care physicians, and midwives had yielded large increases in coverage rates of SIV [22]. Another study by Geraldine and colleagues [23] demonstrated that the determinants on the higher coverage of influenza A (H1N1) vaccine during the 2009 pandemic was significantly associated with the confidence in advice offered by health professionals. Ditsungnoen [24] indicated that increasing healthcare provider awareness of the importance of recommending SIV vaccination to pregnant women could directly increase the likelihood of vaccine acceptance and the coverage would be much higher. Wiley [25] indicated that even women who have safety concerns about the vaccine still indicate that they would accept it if the provider recommended. However, quite a few healthcare providers are hesitant to provide a strong opinion or a recommendation to their patients on vaccination, often due to their lack of the confidence in vaccine safety and fear of the consequences of liability if anything goes wrong [26,27]. Physicians prefer pregnant women to take their own responsibility and decide for themselves. Given perceived fears about SIV among our participants, and the significant association detected between physician recommendations and vaccine acceptance, we underscored the need to encourage health providers to discuss SIV vaccination with their pregnant patients. Their help might be solicited in advocating and recommending vaccine uptake, as well as in dispelling any myths and fears about SIV that pregnant women or their families might harbor.

This study had several limitations. First, this study was implemented in six districts which were not selected randomly, therefor, our results might have selection bias and would have a negative impact on the generalizability to all pregnant women in Zhejiang province. Second, this survey was conducted only in public hospitals, and therefore the sample might not be representative of pregnant women who receive ANCs at private hospitals or did not receive ANCs. Third, this survey only used a closed ended questionnaire rather than included focus group interviews or open ended questions, which would have given an in-depth review on pregnant women’s health beliefs. Forth, this study only evaluated the willingness to accept SIV of the participants but did not follow up their SIV vaccination status. Hence, the actual coverage of SIV of the enrolled pregnant women could not be estimated. 

## 5. Conclusions

In this study, 76% of pregnant women were willing to get seasonal influenza vaccine (SIV) vaccinations during their current pregnancy. Poor knowledge and negative attitude towards SIV was associated with the poor acceptance of SIV. Furthermore, health providers’ recommendations were important to pregnant women’s acceptance of SIV. These findings suggest that health education and direct communication strategies on SIV vaccination and influenza infection through antenatal healthcare providers are necessary to improve the acceptance of SIV vaccination among the pregnant women.

## Figures and Tables

**Table 1:** Descriptive demographic characteristics of pregnant women by acceptance of SIV vaccination, in Zhejiang province, 2014.

Variable		Total (%)	Intention to Accept SIV Vaccination		*χ*^2^	*p*
		*n* = 1252	Yes (%), *n* = 955	No (%), *n* = 297		
Age (year)	<20	114 (9.11)	63 (6.60)	51 (17.17)	98.803	<0.001
	20–30	766 (61.18)	656 (68.69)	110 (37.04)		
	≥31	372 (29.71)	236 (24.71)	136 (45.79)		
Immigration status	Migrant	673 (53.75)	443 (46.39)	230 (77.44)	87.881	<0.001
	Resident	579 (46.25)	512 (53.61)	67 (22.56)		
Education level	≤Junior high school	119 (9.50)	55 (5.76)	64 (21.55)	125.60	<0.001
	Senior high school or technical school	378 (30.19)	248 (25.97)	130 (43.77)		
	≥College	755 (60.30)	652 (68.27)	103 (34.68)		
Occupation	Housewife	113 (9.03)	68 (7.12)	45 (15.15)	15.72	<0.001
	Employed	1139 (90.97)	877 (91.83)	262 (88.22)		
Monthly household income per capita	<800 CNY	247 (19.73)	139 (14.55)	108 (36.36)	69.173	<0.001
	800–1500 CNY	622 (49.68)	512 (53.61)	110 (37.04)		
	>1500 CNY	383 (30.59)	304 (31.83)	79 (26.60)		
Number of children *	0	635 (50.72)	422 (44.19)	213 (71.73)	79.723	<0.001
	1	452 (36.10)	406 (42.51)	46 (15.49)		
	≥2	165 (13.18)	127 (13.30)	38 (12.79)		

*: excluded the unborn child of the current pregnancy, SIV: seasonal influenza vaccine.

**Table 2:** Comparison of the proportion of pregnant women who agreed with various health beliefs by acceptance of SIV, in Zhejiang province, 2014.

HBM Construct	Item	Response Statement	Intention to Accept SIV Vaccination		*χ*^2^	*p*
			Yes (%), *n* = 955	No (%), *n* = 297		
Susceptibility	Are you concerned about getting influenza	Very/moderate concerned	622 (65.13)	83 (27.95)	127.33	<0.001
		Not concerned	333 (34.87)	214 (72.05)		
	Are you concerned about unborn baby getting influenza	Very/moderate concerned	680 (71.20)	95 (31.99)	147.74	<0.001
		Not concerned	275 (28.80)	202 (68.01)		
Severity	If a pregnant woman gets influenza, she is more likely to have severe illness	Agree	492 (51.52)	155 (52.19)	0.041	0.84
		Not sure or disagree	463 (48.48)	142 (47.81)		
	If a pregnant woman gets influenza, the illness could harm her unborn baby	Agree	726 (76.02)	157 (52.86)	58.45	<0.001
		Not sure or disagree	229 (23.98)	140 (47.14)		
Barriers	SIV can cause a person to get sick with influenza	Agree	164 (17.17)	105 (35.35)	44.39	<0.001
		Not sure or disagree	791 (82.83)	192 (64.65)		
	SIV is not safe during pregnancy	Agree	142 (14.87)	89 (29.97)	34.32	<0.001
		Not sure or disagree	813 (85.13)	208 (70.03)		
	SIV is not an effective way to prevent a pregnant woman from getting influenza	Agree	155 (16.23)	90 (30.30)	28.51	<0.001
		Not sure or disagree	800 (83.77)	207 (69.70)		
Benefits	Giving SIV to a pregnant woman will benefit her fetus and new born baby	Agree	738 (77.28)	184 (61.95)	27.41	<0.001
		Not sure or disagree	217 (22.72)	113 (38.05)		
	Getting SIV during pregnancy is a benefit for the pregnant woman	Agree	764 (80.00)	206 (69.36)	14.70	<0.001
		Not sure or disagree	191 (20.00)	91 (30.64)		
	SIV could protect the baby during the first months of life	Agree	458 (47.96)	136 (45.79)	0.43	0.51
		Not sure or disagree	497 (52.04)	161 (54.21)		
Cues to action	If physician/nurse recommended SIV I would get vaccinated	Agree	889 (93.09)	221 (74.41)	78.60	<0.001
		Not sure or disagree	66 (6.91)	76 (25.59)		
	If relative recommended SIV I would get vaccinated	Agree	383 (40.10)	119 (40.07)	0.001	0.98
		Not sure or disagree	572 (59.90)	178 (59.93)		

HBM: health belief model.

**Table 3:** Bivariate and multivariable analysis indicating associations between HBM constructs and the acceptance of SIV vaccination among pregnant women, in Zhejiang province, 2014.

HBM Construct		Intention to Accept SIV Vaccination		COR (95%CI)	AOR (95%CI)
		Yes (%), *n* = 955	No (%), *n* = 297		
Susceptibility	Low	115 (12.04)	67 (22.56)	Reference	Reference
	Moderate	263 (27.54)	103 (34.68)	1.37 (1.16–1.62)	1.08 (0.92–1.30)
	High	577 (60.42)	127 (42.76)	2.23 (1.92–2.70)	1.75 (1.36–2.08)
Severity	Low	127 (13.30)	87 (29.29)	Reference	Reference
	Moderate	306 (32.04)	98 (33.00)	1.08 (0.86–1.27)	1.01 (0.86–1.14)
	High	522 (54.66)	112 (37.71)	1.83 (1.42–2.55)	1.62 (1.25–1.95)
Barriers	Low	604 (63.25)	125 (42.09)	Reference	Reference
	Moderate	277 (29.01)	100 (33.67)	0.86 (0.73–0.95)	0.91 (0.85–1.14)
	High	74 (7.75)	72 (24.24)	0.62 (0.48–0.79)	0.76 (0.62–0.94)
Benefits	Low	82 (8.59)	71 (23.91)	Reference	Reference
	Moderate	280 (29.32)	99 (33.33)	1.69 (1.55–2.12)	1.85 (1.65–2.32)
	High	593 (62.09)	127 (42.76)	1.91 (1.72–2.20)	1.97 (1.76–2.21)
Cues to action	Low	76 (7.96)	84 (28.28)	Reference	Reference
	Moderate	237 (24.82)	101 (34.01)	1.53 (1.17–1.74)	1.32 (1.10–1.64)
	High	642 (67.23)	112 (37.71)	2.33 (1.85–2.62)	2.03 (1.70–2.69)

COR: crude odds ratio; AOR: adjusted odds ratio with other demographic variables.
